# Long-chain omega-3 fatty acids and the brain: a review of the independent and shared effects of EPA, DPA and DHA

**DOI:** 10.3389/fnagi.2015.00052

**Published:** 2015-04-21

**Authors:** Simon C. Dyall

**Affiliations:** Faculty of Health and Social Sciences, Bournemouth UniversityBournemouth, UK

**Keywords:** aging, Alzheimer’s disease, eicosapentaenoic acid, docosahexaenoic acid, docosapentaenoic acid, omega-3 fatty acids, Parkinson’s disease

## Abstract

Omega-3 polyunsaturated fatty acids (PUFAs) exhibit neuroprotective properties and represent a potential treatment for a variety of neurodegenerative and neurological disorders. However, traditionally there has been a lack of discrimination between the different omega-3 PUFAs and effects have been broadly accredited to the series as a whole. Evidence for unique effects of eicosapentaenoic acid (EPA), docosahexaenoic acid (DHA) and more recently docosapentaenoic acid (DPA) is growing. For example, beneficial effects in mood disorders have more consistently been reported in clinical trials using EPA; whereas, with neurodegenerative conditions such as Alzheimer’s disease, the focus has been on DHA. DHA is quantitatively the most important omega-3 PUFA in the brain, and consequently the most studied, whereas the availability of high purity DPA preparations has been extremely limited until recently, limiting research into its effects. However, there is now a growing body of evidence indicating both independent and shared effects of EPA, DPA and DHA. The purpose of this review is to highlight how a detailed understanding of these effects is essential to improving understanding of their therapeutic potential. The review begins with an overview of omega-3 PUFA biochemistry and metabolism, with particular focus on the central nervous system (CNS), where DHA has unique and indispensable roles in neuronal membranes with levels preserved by multiple mechanisms. This is followed by a review of the different enzyme-derived anti-inflammatory mediators produced from EPA, DPA and DHA. Lastly, the relative protective effects of EPA, DPA and DHA in normal brain aging and the most common neurodegenerative disorders are discussed. With a greater understanding of the individual roles of EPA, DPA and DHA in brain health and repair it is hoped that appropriate dietary recommendations can be established and therapeutic interventions can be more targeted and refined.

## Introduction

There is mounting evidence supporting the beneficial effects of an increased intake of omega-3 polyunsaturated fatty acids (PUFAs) in a variety of neurodegenerative and neurological conditions (Dyall and Michael-Titus, 2008; Dyall, 2010; Denis et al., 2015). However, comparing results between studies has traditionally been hampered by a lack of discrimination between the different omega-3 PUFAs and results are typically attributed the omega-3 PUFAs as a whole (Dyall, 2011). There is accumulating evidence that certain effects may be unique and specific to individual omega-3 PUFAs and there can no longer be an assumption of equivalence in either mechanism of action or function. This issue is particularly important when pooling studies in systematic reviews and meta-analyses. For example, two recent meta-analyses into the effects of EPA and DHA in depression reviewed predominantly the same studies, but produced substantially different outcomes (Sublette et al., 2011; Bloch and Hannestad, 2012). Bloch and Hannestad found only small, non-significant benefits, with no significant differences between EPA and DHA (Bloch and Hannestad, 2012). Whereas Sublette and coworkers used a mixed-effect model, separating the treatments by EPA content, and found that only supplements with the proportion of EPA ≥ 60% of the total EPA and DHA content in a dose range of 200–2200 mg EPA in excess of DHA effective against primary depression (Sublette et al., 2011). Although a number of methodological differences between the meta-analyses may have contributed to the different outcomes (Lin et al., 2012b); this incongruity between findings highlights the importance of considering the omega-3 PUFAs as biologically distinct molecules. Furthermore, although current evidence suggests that EPA and DHA are the predominant biological omega-3 PUFAs, the bioactive effects of docosapentaenoic acid (DPA, 22:5n-3) are gaining recognition in the literature (Kaur et al., 2011).

A number of excellent reviews have discussed the complementary and divergent effects of the different omega-3 PUFAs both at the fundamental level in terms of cell signaling and function (Gorjão et al., 2009; Russell and Bürgin-Maunder, 2012), and also in diseases such as cancer, insulin resistance, and cardiovascular disease (Anderson and Ma, 2009; Mozaffarian and Wu, 2012). The aim of this current article is to review the effects of the different long-chain omega-3 PUFAs in the brain in normal aging and neurodegenerative disorders. Although the short-chain omega-3 PUFAs, α-linolenic acid (ALA, 18:3n-3) and stearidonic acid (SDA, 18:4n-3) have shown potential health benefits (Barceló-Coblijn and Murphy, 2009; Whelan, 2009), these have yet to be sufficiently explored in the context of central nervous system (CNS) disorders, and therefore this review will be restricted to EPA, DPA and DHA.

## EPA, DPA and DHA Metabolism

**Figure 1:**
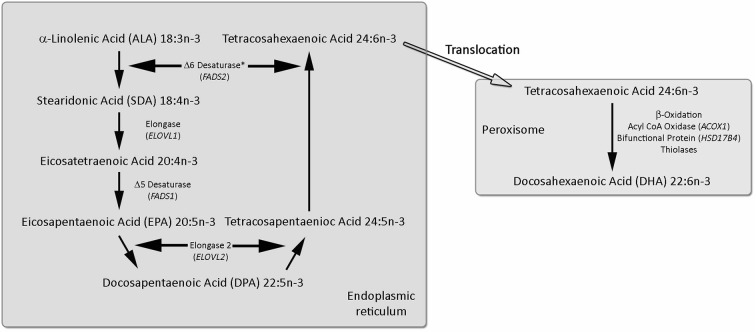
**Synthesis of EPA, DPA and DHA from ALA**. The longer chain omega-3 polyunsaturated fatty acids (PUFAs) are synthesized from ALA by a progressive series of enzymatic desaturation and chain elongation steps, initially in the endoplasmic reticulum. In the final stage tetracosahexaenoic acid (24:6n-3) is translocated to the peroxisome and is shorted by one cycle of the β-oxidation pathway to form DHA (22:6n-3). For further details refer to the text. Figure adapted from Dyall and Michael-Titus (2008).

The omega-3 PUFA series begins with ALA with the other omega-3 PUFAs derived from ALA via a series of desaturation, elongation and ultimately β-oxidation reactions (Moore et al., 1995; Gregory et al., 2011), summarized in Figure 1. The pathway begins with the desaturation of ALA to SDA by Δ6 desaturase (encoded by *FADS2* genes), which is a rate limiting step. This is followed by elongation (*ELOVL1* gene) to eicocosatetraenoic acid (20:4n-3). Desaturation by Δ5 desaturase (*FADS1* gene) produces EPA and EPA is then elongated by elongase-2 (*ELOVL2* gene) first to DPA and then tetracosapentaenoic acid (24:5n-3). Tetracosapentaenoic acid then undergoes a second Δ6 desaturation to produce tetracosahexaenoic acid (24:6n-3). These initial steps occur in the endoplasmic reticulum; however, the final stage of DHA synthesis occurs in the peroxisome following translocation. In the peroxisome 24:6n-3 is shortened to DHA (22:6n-3) by a single round of β-oxidation by the action of acyl-coenzyme-A oxidase (*ACOX1* gene), _D_-bifunctional enzyme (*HSD1784* gene) and then peroxisomal thiolases.

Although this pathway is now well characterized, the efficiency and kinetics of conversion in humans has been somewhat harder to establish. There are limited tissues readily available for analysis, so blood components are typically analyzed, which although showing strong correlations with peripheral tissues, may not reflect the composition of the CNS (Tu et al., 2013). Furthermore, it has long been known that blood components differ in their metabolism and incorporation of EPA, DPA and DHA (Brown et al., 1991). In an early short-term fish oil supplementation study erythrocyte EPA was a stronger indicator of omega-3 PUFA intake than DPA or DHA, potentially due to differential incorporation into the membrane phospholipids. EPA content appears to be largely determined by exchange with plasma lipoproteins, whereas DPA and DHA are more enriched in the inner membrane and are therefore more influenced by erythrocyte turnover (Brown et al., 1991). More recently these different incorporation rates and efficiencies have also been shown in other blood components (Metherel et al., 2009; Miller et al., 2013). For example, when participants were given 8 g of pure EPA or DPA over a 7 day period, EPA supplementation significantly increased EPA in erythrocytes and plasma cholesterol esters and phospholipids, but not plasma triacylglycerols. Whereas, DPA supplementation significantly increased DPA in plasma triacylglycerols and phospholipid fractions, but not erythrocyte or plasma cholesterol esters (Miller et al., 2013). These differential efficiencies of incorporation are not be restricted to blood and are also seen in adipose tissue (Katan et al., 1997). Recent studies investigating the differences in metabolism between EPA, DPA and DHA are summarized in Table 1, where it appears a potential role for DPA may be to act as a reservoir for EPA and DHA. Earlier studies have been extensively reviewed by Kaur et al. (2011).

**Table 1:** Summary of the metabolism, neuroprotective and anti-inflammatory effects of DPA*.

Experimental model	Main outcomes	Reference
	Metabolism
Sprague Dawley rats	DPA increased DPA in all tissues and DHA in the liver. DPA also partly retro-converted to EPA in liver, adipose tissue, heart and skeletal muscle.	Kaur et al. (2010)
Sprague Dawley rats	DPA increased DPA in heart and liver and increased EPA content with the retro-conversion particularly pronounced in the kidney.	Holub et al. (2011)
Wistar rats	DPA and DHA β-oxidized significantly less than EPA at 6 h, and higher incorporation of DPA and DHA in skeletal muscle and heart than EPA.	Kaur et al. (2013)
Sprague Dawley rats	Greater excretion of DPA in feces than EPA. EPA and DPA similarly increased EPA, DPA and total long chain omega-3 PUFAs in the liver.	Ghasemi Fard et al. (2014)
Healthy females (age 21–30 years)	After 4 days supplementation DPA increased EPA, DPA and DHA content of plasma or RBC lipids, whereas EPA only increased EPA content.	Miller et al. (2013)
Healthy females (age 20–30 years)	EPA and DPA metabolized differently postprandially. DPA significantly decreased chylomicronemia compared to EPA.	Linderborg et al. (2013)
	Neuroprotection
Young (3–4 months) and old (20–22 months) rats	EPA increased cortical tissue DPA and DHA in young and old rats and EPA in old rats, whereas DPA increased DPA in young and old rats and DHA in young rats. EPA and DPA similarly down-regulated age-related microglial activation, decreased activation of sphingomyelinase and caspase 3 and restored long-term potentiation and improved spatial memory in the aged rats.	Kelly et al. (2011)
	Inflammation
Mice neutrophils and human macrophages	DPA-derived PD1_n-3 DPA_ significantly reduced neutrophil recruitment during peritonitis of mice and stimulated macrophage phagocytosis and clearance of apoptotic human neutrophils, both to a similar extent to DHA-derived PD1.	Aursnes et al. (2014)
Human macrophages	DPA-derived Mar1_n-3 DPA_ stimulated macrophage phagocytosis and clearance of apoptotic human neutrophils to a similar extent to that of DHA-derived Mar1.	Tungen et al. (2014)

* For a detailed review of the metabolism and biological effects of DPA prior to 2011 see Kaur et al. (2011).

It has also been demonstrated that variations in genes involved in the biosynthesis and metabolism of omega-3 PUFAs affects their blood levels. In a study of 8866 participants from European ancestry single nucleotide polymorphisms (SNPs) in desaturase genes *FADS1* (Δ5 desaturase) and *FADS2* (Δ6 desaturase) were associated with higher ALA and lower EPA and DPA plasma phospholipid levels, suggesting different rates of conversion (Lemaitre et al., 2011). Similarly, higher levels of EPA and DPA and lower DHA were associated with SNPs in *ELOVL2* (elongase 2). Furthermore, those with the apoE-ε4 (ApoE-ε4) polymorphism show a lower response to EPA and DHA supplementation (Plourde et al., 2009), and disturbed DHA metabolism, such that DHA has a significantly decreased half-life and enhanced β-oxidation (Chouinard-Watkins et al., 2013). The ApoE-ε4 allele is the major genetic risk factor for the development of late onset AD (Hauser and Ryan, 2013), and importantly ApoE-ε4 carriers seem not to be protected against AD when consuming fish (Chouinard-Watkins et al., 2013). Gender also appears to differentially affect long-chain omega-3 PUFA levels, whereby females have significantly lower blood levels of DPA and EPA, but significantly higher DHA than males (Metherel et al., 2009).

These individual variations in response also extend to changes in the levels of omega-6 and omega-3 PUFA-derived metabolites (Nording et al., 2013). In this detailed lipidomics analysis 12 subjects were given 1.9 g/day EPA and 1.5 g/day DHA for 6 weeks. There were some shared responses to the supplementation with the participants, such as significantly decreased plasma triacylglycerols, very low density lipoproteins (VLDLs) and chylomicron particles. However, there was also a very high degree of inter-individual variability in the effects of supplementation on the production of omega-6 and omega-3 PUFA-derived metabolites. For example, production of the EPA lipoxygenase (LOX) metabolite 12-hydroxyeicosapentaenoic acid (12-HEPE) varied between subjects from an 82% decrease to a 5,000% increase.

However, in spite of these individual variations, studies into the effects of omega-3 PUFA supplementation on blood composition in normal healthy adults consuming Western diets do show some consistent responses (Arterburn et al., 2006; Brenna et al., 2009). ALA supplementation induces significant increases in EPA and DPA content, but has negligible effects on DHA content in plasma or blood cells (in the order of 1% in infants and much lower in adults). EPA supplementation increases blood EPA and DPA levels to an approximately 15-fold greater extent than ALA, but also does not affect DHA. Only pre-formed DHA supplementation significantly increases blood DHA levels (Brenna et al., 2009). Consequently the most consistent way to increase EPA, DPA or DHA blood content is to supplement with that particular PUFA. However, care does needs to be taken when comparing between studies and extrapolating from blood to CNS levels.

## EPA, DPA and DHA Metabolism in the Brain

The brain has a unique fatty acid composition with high levels of palmitate (16:0), the omega-6 PUFA arachidonic acid (AA, 20:4n-6), and DHA, but low levels of other omega-3 PUFAs, especially EPA (Crawford et al., 1976; Brenna and Diau, 2007). Indeed, brain EPA levels are typically 250–300 times lower than DHA (Chen et al., 2009). Thus, DHA is quantitatively the most important omega-3 PUFA in the brain. In addition to differences in tissue levels of EPA, DPA and DHA, there are also differences in their phospholipid location within the brain, such that DHA and DPA are predominantly enriched in phosphatidylethanolamine (PE) and phosphatidylserine (PS), whereas EPA appears preferably esterified to phosphatidylinositol (PI; Chen et al., 2009). Indeed, EPA shares this proclivity for PI with the AA, which is also highly enriched in PI (Lee and Hajra, 1991), and may be related to their equivalent chain length.

The endogenous synthesis of EPA, DPA and DHA are low within the brain compared with uptake from the plasma unesterified fatty acid pool (Demar et al., 2005, 2006), suggesting that the brain maintains levels via the uptake from dietary and/or liver sources in plasma. Using an *in situ* cerebral perfusion competition assay, EPA and DHA were found to enter the brain at similar rates, and therefore appear to cross the blood brain barrier by simple diffusion (Ouellet et al., 2009), and presumably DPA acts in the same manner. The low levels of EPA are maintained by multiple mechanisms including β-oxidation, decreased incorporation, elongation to DPA and lower phospholipid recycling (Chen et al., 2013; Kaur et al., 2013).

It is currently unclear why brain phospholipids are specifically enriched in DHA and low in DPA and EPA. As described above, biosynthesis of DHA from DPA involves elongation and desaturation, followed by translocation to the peroxisome for β-oxidation. This is not only more energetically costly, but also introduces PUFAs into the membrane with much greater potential for peroxidation. Furthermore, this specific DHA enrichment is conserved across species (Crawford et al., 1976; Farkas et al., 2000), suggesting that there a highly specific requirement for DHA in the neuronal membrane.

## Effects of EPA, DPA and DHA on Neuronal Membrane Properties

A variety of membrane effects have been shown with DHA, including modulating key biophysical properties such as acyl chain order, membrane fluidity, phase behavior, compression, permeability, fusion, flip-flop and protein activity (Stillwell and Wassall, 2003; Stillwell et al., 2005), and driving the creation of cholesterol-depleted domains (Wassall and Stillwell, 2008). However, there have been few direct comparisons of the membrane properties of EPA, DPA and DHA, to explain this indispensable role, although recently DHA and EPA were shown to partition differently in raft and non-raft domains in membranes (Williams et al., 2012). DHA had a much greater tendency to accumulate into sphingomyelin/cholesterol-rich lipid rafts than EPA, and therefore had a much greater potential to affect cell signaling by modify the composition of these lipid rafts. A further intriguing recent hypothesis for the indispensable nature of DHA suggests that the unique structure of DHA allows for the quantum transfer and communication of π-electrons across the membrane (Crawford et al., 2013). This hypothesis offers an explanation for the precise depolarisation of retinal membranes and the cohesive, organized neural signaling essential for higher intelligence.

An important role for DHA in the membrane is to modulate the synthesis of PS, as the accumulation and biosynthesis of PS in neuronal tissues is sensitive to the level of membrane DHA (Garcia et al., 1998) and 18:0, DHA is the most abundant PS species in the brain (Kim et al., 2014a). The biosynthesis of PS occurs from pre-existing phosphatidylcholine (PC) or PE via serine base exchange, and DHA at the *sn*-2 position on PC is more favorably converted to PS (Kim et al., 2004). Increasing the PS content of neuronal membranes may positively affect neuronal survival via the PI 3-kinase/Akt signaling pathway (Akbar et al., 2005). However, when directly compared, dietary EPA and DHA similarly increase brain phospholipid levels (Cansev and Wurtman, 2007). Gerbils fed EPA or DHA (300 mg/kg/day by oral gavage) had significantly increased brain levels of PE, PS and PI compared to animals on the control diet. The DHA group also had significantly increased PC levels. These effects were accompanied by increases in the levels of pre and post-synaptic proteins, syntaxin-3, PSD-95 and synapsin-1, and were not seen with following AA treatment. In addition to the accumulation of phospholipids DHA has further effects on membrane phospholipid composition. A detailed lipidomics analysis found that rats supplemented with 10 mg/day of DHA for 8 weeks induced multiple changes in phospholipid composition, including the production of new PS and PI species (Little et al., 2007).

The indispensable nature of DHA in neuronal membranes has also been shown by omega-3 PUFA dietary deficiency studies, whereby reduction of brain phospholipid DHA consistently produces reciprocal increases in the levels of DPAn-6 (Enslen et al., 1991; Contreras et al., 2001). Since omega-3 and omega-6 PUFAs are recycled independently of each other, this relationship is probably maintained through competition for Δ6D (Contreras et al., 2001). Omega-3 PUFAs competitively inhibit the desaturation of linoleic acid (18:2n-6) by Δ6D (Galli et al., 1971), and therefore subsequent biosynthesis of longer chain omega-6 PUFAs. This reciprocal relationship maintains the degree of phospholipid membrane unsaturation to approximately constant levels, in a process called “homeoviscous compensation” (Garda et al., 1994). However, in spite of this homeoviscous compensation profound and widespread effects are observed when tissue DHA levels are decreased, such as alterations to membrane properties (Eldho et al., 2003), decreases in performance in tasks of spatial memory (Lim et al., 2005), altered enzyme activity and electrophysiological properties (Bourre et al., 1989), and altered neurotransmission (Able et al., 2014; Cardoso et al., 2014).

Overall, these studies highlight the unique and indispensable role of DHA in neuronal membranes, whereas, the only established unique role for EPA is as a precursor to the three series eicosanoids and related peroxy-fatty acids, although specific therapeutic effects have been putatively identified across a range of neurological conditions. Similarly, bioactive roles for DPA are now being described. The remainder of this review will explore the evidence for both specific and complementary effects of EPA, DPA and DHA in the brain.

## EPA, DPA and DHA Derived Lipid Mediators

It is well established that omega-3 PUFA derived lipid mediators play a key role in the inflammatory response. These enzyme-derived mediators are a varied group of oxygenated forms of the C20 and C22 omega-3 PUFAs. The classic lipid mediators include prostaglandins and leukotrienes (LT), and two structurally distinct new classes of mediators have been identified. The first are called “Specialized Pro-resolving Mediators” (SPMs; Bannenberg and Serhan, 2010), and the second are electrophilic fatty acid oxo-derivatives (Groeger et al., 2010; Cipollina et al., 2014).

The classic lipid mediators are a varied group of oxygenated forms, with those derived from the C20 PUFAs, such as EPA, called eicosanoids, whereas those from C22 DPA and DHA called docosanoids. Biosynthesis is catalyzed by three enzyme systems: cyclooxygenase (COX, also known as prostaglandin endoperoxide H synthase or PGHS) and subsequent synthases, LOX and cytochrome P450 mixed-function oxidase enzymes (CYP450; Smith et al., 2000). COX catalyzes the initial oxygenation of non-esterified PUFAs to produce prostaglandin H (PGH), a short-lived intermediate, which is further metabolized to other prostaglandin series (PG), prostacyclins (PGI), thromboxanes (TXA), LT, lipoxins (LX), hydroxy and hydroperoxy fatty acids (Smith et al., 2000). Vertebrates have two principal isoforms of COX: COX-1 and COX-2 (Kulmacz et al., 2003).

In most tissues COX-1 is constitutively expressed, whereas COX-2 is inducible. However, in specialized cell types such as the neurones in the CNS, constitutive COX-2 expression is observed in the cortex and allocortical structures, such as the hippocampus and amygdala (Breder et al., 1995). COX-2 is particularly enriched in the hippocampus and cortex (Yamagata et al., 1993), where expression is localized in the postsynaptic dendritic spines (Kaufmann et al., 1996), suggesting a role in neural activity and activity-dependent plasticity. COX-1 and -2 have similar structures, although COX-1 has a smaller active site (Rowlinson et al., 2000). This size restriction imposes a substrate specificity on COX-1 for C20 fatty acids (Kulmacz et al., 2003), and is unable to oxygenate DHA (Corey et al., 1983). The COX-2 active site is about 20% larger (Smith et al., 2000) and this enzyme is consequently able to metabolize a larger range of substrates, with differing acyl chain length and degree of unsaturation, potentially including DPA and DHA.

The SPMs are a rapidly expanding class of autacoid molecules involved in the active resolution of inflammation, and are produced through COX and LOX pathways (Serhan et al., 2008). EPA produces E-series resolvins (RvE), whereas DHA produces docosanoids, such as protectins, D-series resolvins (RvD) and maresins (MaR; Serhan et al., 2015). Recently analogous mediators to DHA-derived D-series resolvins, protectins and MaRs have been identified from DPA, and include RvD1_n-3 DPA_, MaR1_n-3 DPA_ and related products (Dalli et al., 2013). PD1_n-3 DPA_ and MaR1_n-3 DPA_ have recently been synthesized and demonstrate potent anti-inflammatory and pro-resolving properties, with comparable actions to EPA and DHA-derived resolvins in murine models of acute inflammation and human leukocytes (Aursnes et al., 2014; Tungen et al., 2014). The rapidly expanding repertoire of oxygenated metabolites from EPA, DPA and DHA and the similarity between their isomers has the scope for confusion in assigning biological properties, and it has been suggested that care needs to be taken when interpreting the literature (Balas et al., 2014).

The SPMs act via a series of cell-type specific receptors, which due to space restraints are beyond the scope of this review; however, the reader is directed to a number of excellent reviews by Dr. Charles Serhan, whose laboratory has led research in this area (Serhan et al., 2009; Bannenberg and Serhan, 2010; Dalli et al., 2013; Serhan and Chiang, 2013). However, by way of a brief example, RvE1 binds the orphan receptor ChemR23, and BLT_1_, a leukotriene B_4_ receptor, whereas RvD1 binds GPR32 and ALX, a lipoxin A_4_ receptor. This specificity may allow for specific effects of EPA, DPA and DHA-derived SPMs. The best characterized SPM in terms of nervous system protection is (neuro)protectin D1 ((N)PD1, 10*R*-17*S*-dihydroxy-docosahexaenoic acid), which is biosynthesized in response to injury and may have therapeutic potential in a wide range of neurological conditions (Bazan et al., 2011b; Bazan, 2013). The unique neuroprotective potential of EPA and DPA-derived SPMs remains to be elucidated.

**Figure 2:**
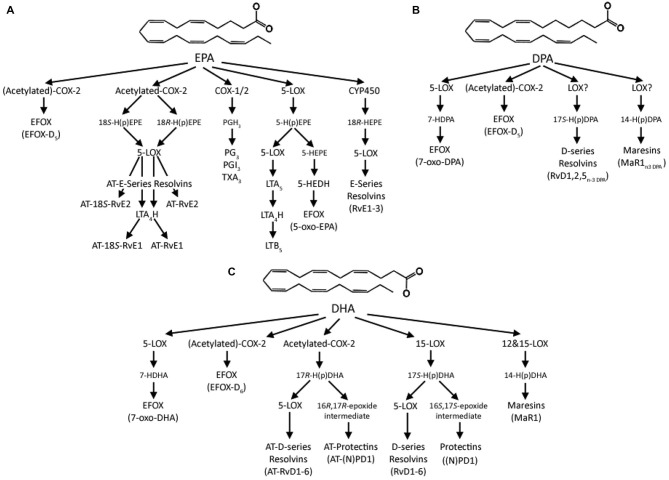
**Summary of the lipid mediators derived from (A) EPA, (B) DPA and (C) DHA**. In the classical “canonical” pathway EPA is initially converted to the intermediate prostaglandin G_2_ (PGG_2_) by either COX-1 or -2 and then enzymatically to the 3 series prostaglandins, prostacylcins or thromboxanes. EPA can also be converted by 5-lipoxygenase (LOX) to 5-hydroperoxyeicosapenataenoic acid (5-H(p)EPE), which can then either be converted by 5-LOX to leukotriene A_5_ (LTA_5_) and then by Leukotriene A_4_ Hydrolase (LTA_4_) to leukotriene B_5_ (LTB_5_) or to 5-hydroxyeicosapentaenoic acid (5-HEPE), which is then converted into 5-oxo-EPA by 5-hydroxyeicosanoid dehydrogenase (5-HEDH). EPA can also be sequentially converted by cytochrome P450 (CYP450) enzymes to 18*R*-hydroxyeicosapentaenoic acid (18*R*-HEPE) and then by 5-LOX to E-series resolvins (RvE). COX-2 can also convert EPA to the electrophilic fatty acid oxo-derivative electrophilic fatty acid oxo-derivates (EFOX)-D_5_, in a process enhanced by aspirin acetylation of COX-2. Aspirin acetylation of COX-2 also produces 18*S*- and 18*R*-hydroperoxyeicosapentaenoic acids (18*S-*, or 18*R*-HETE) from EPA, which are either converted by 5-LOX to aspirin-triggered 18*S*-resolvin E1 and resolvin E1 (AT-18*S*-RvE1 and AT-RvE1), respectively, or through an extra step by LTA_4_H to AT-18*S*-RvE2 and AT-RvE2. Analogous series of resolvins, maresins, and EFOXs produced from DPA to those from DHA have recently been identified; however, the nature of the enzymatic conversions remains to be elucidated. DHA is converted to 17*S*-hydroperoxydocosahexaenoic acid (17*S*-H(p)DHA) by 15-LOX, which is converted by 5-LOX to D-series resolvins (RvD), or enzymatically hydrolysed to (neuro)protectin D1 ((N)PD1. DHA can also be converted by 12 or 15-LOX via 14-hydroperoxydocosahexaenoic acid (14-H(p)DHA) to the maresins. DHA can also be converted by 5-LOX to 7-hydroxydocosahexaenoic acid (7-HDHA) and then by a dehydrogenase to 7-oxo-DHA, with 5-HEDH a likely candidate, or by COX-2 to EFOX-D_6_, which is enhanced by aspirin acetylation. Acetylation also produces 17*R*-hydroperoxyDHA, which can then be converted to aspirin triggered resolvins and protectins.

A further novel class of anti-inflammatory lipid mediators derived from EPA, DPA and DHA has recently been described (Groeger et al., 2010; Cipollina et al., 2014). These are electrophilic fatty acid oxo-derivates (EFOX) synthesized by COX-2 and 5-LOX, and include 5-oxo-EPA, 7-oxo-DPA and 7-oxo-DHA, from EPA, DPA and DHA, respectively. In the first charactrerisation of their actions EFOXs were shown to have a wide range of anti-inflammatory actions, including acting as agonists of the nuclear transcription factor, peroxisome proliferator-activated receptor gamma (PPARγ), activating Nrf2-dependent antioxidant reponses and inhibiting cytokine production and inducible nitric oxide expression in activated macrophages (Groeger et al., 2010). Importantly, dietary supplementation with 1 g EPA and 0.4 g DHA per day for 4 months significantly increases the formation of 5-oxo-EPA and 7-oxo-DHA (Cipollina et al., 2014), and consitent with the formation of resolvins from DHA (Serhan et al., 2002), aspirin acetylation of COX-2 also significantly increases production of EFOXs. Although independent roles of the different EFOXs have yet to be described, these observations suggest these newly reported lipid mediators needs to be considered when assiging anti-inflammatory and pro-resolving effects to EPA, DPA and DHA. The multiple lipid mediators derived from EPA, DPA and DHA are summarized in Figure 2.

## Effects of EPA, DPA and DHA on Brain Plasticity

### Neurite Outgrowth and Synaptogenesis

The synapse and nerve growth cone are two structures that play key roles in development and nervous system repair, furthermore, slow neurodegenerative processes in the CNS are accompanied by significant damage to neurite (Robson et al., 2010). The effects of EPA and DHA, but not DPA have been studied on neurite outgrowth and synaptogenesis in a variety of cell types and stages of development. Early studies focused on DHA, and found beneficial effects on neurite outgrowth in terms of overall length and complexity of outgrowth in rat pheochromocytoma-12 (PC-12) cells (Ikemoto et al., 1997), rat embryonic hippocampal primary cultures (Calderon and Kim, 2004) and rat embryonic cortical neurons (Cao et al., 2005). In addition to enhanced neurite outgrowth, DHA also promotes synaptogenesis and synaptic expression of synapsin, and glutamate receptors in rat hippocampal neurones (Cao et al., 2009). Work in our laboratory directly compared the neurite-promoting effects of DHA with EPA in primary sensory neuronal cultures from young (post-natal day 3 and 9), adult (2–4 months) and aged (18–20 month) rats (Robson et al., 2010). Both EPA and DHA increased neurite outgrowth in the developmental stages; however, only DHA produced positive effects in the tissue from aged rats, which possess a significantly lower degree of plasticity than tissues from immature animals.

### Neurogenesis

Adult neurogenesis is the process by which new neurons are generated from neural stem cells and progenitor cells and has been consistently shown to occur in two areas of the adult brain, the subventricular zone of the lateral ventricles and the subgranular layer of the hippocampal dentate gyrus (Ehninger and Kempermann, 2008). Aging is the greatest negative regulator of neurogenesis (Kuhn et al., 1996), and there is a strong correlation between age-related impairments in hippocampal-dependent memory tasks and the decline in neurogenesis (Drapeau et al., 2003). Conversely, increased neurogenesis in reported in rodents following ischemia (Takagi et al., 1999), stroke (Darsalia et al., 2005) and after seizures (Parent et al., 1997). These increases in neurogenesis may be an attempt at brain self-repair and raises the intriguing possibility that enhancing neurogenesis and the subsequent survival of new neurons may have significant therapeutic potential.

*In vitro* DHA has consistently been shown to promote the differentiation of neural stem cells into neurons (Kawakita et al., 2006; Katakura et al., 2009); however, comparisons of the pro-neurogeneic effects of different omega-3 PUFAs have only recently begun to be explored in the literature. In the first direct comparison 1 µM DHA and EPA were both reported to enhance differentiation of neural stem cells to a similar extent (Katakura et al., 2013). However, they had divergent effects on the transcription factors involved in regulating the cell cycle. EPA significantly increased the levels of Hes1, whereas with DHA levels were significantly decreased. Hes1 is a repressor type transcription factor, which inhibits neuronal differentiation and enhances proliferation in neural stem cells (Katakura et al., 2013). Also, EPA, but not DHA, significantly increased Hes6, which acts in a positive-feedback loop with Hes1 to promote neuronal differentiation. Preliminary work in our laboratory also suggests divergent effects of EPA and DHA on neurogenesis; however, in this study 10 nM EPA significantly increased proliferation, whereas with 10 nM DHA it was decreased, consistent with a role in differentiation (Mandhair et al., 2013). It may be that the effects of EPA and DHA in regulating neural stem cell fate are based on a fine balance between their respective effects at basic helix-loop-helix transcription factors.

Although the addition of EPA and DHA show regulatory effects on neural stem cell fate, it is currently unclear whether these are direct effects of EPA and DHA or their metabolites. The DHA endocannabinoid-like metabolite, N-docosahexanoylethanolamine (DHEA, also known as synaptamide) induces neuronal differentiation of neural stem cells by activation of protein kinase A (PKA)/cAMP response element binding protein (CREB; Rashid et al., 2013). Furthermore, the oxygenated DHA-derivative NPD1 has also been shown to promote neuronal differentiation of embryonic stem cells (Yanes et al., 2010). Overall however, these results suggest that the regulatory effects of EPA and DHA directing neural stem cell fate are mediated via divergent effects at transcription factors and potentially different signaling pathways, and may be at least in part mediated by their enzymatic conversion to bioactive mediators.

## Normal Brain Aging and Alzheimer’s Disease

Many widespread changes characterize normal brain aging, such as increased oxidative stress (Perluigi et al., 2014), mitochondrial dysfunction and alterations in energy metabolism (Ames, 2004) and damage to DNA (Canugovi et al., 2013). There are also age-related structural changes including a reduction in brain volume and weight (Anderton, 2002) and altered membrane lipid content (Svennerholm et al., 1997). The aging brain is more prone to the development of neurodegenerative diseases, such as Alzheimer’s disease (AD), and with the slow developing preclinical phases of these diseases it may be difficult to distinguish what are age-related changes and what are effects of the undetected disease (Fjell et al., 2014). AD is the most common form of dementia in the older person, with a prevalence of about 4.4% of the population over 65 years of age (Ward et al., 2012) and affects cognitive function, mood and behavior (Selkoe et al., 2012). The characteristic pathological features of AD are neurofibrillary tangles, which are mainly composed of aggregates of hyperphosphorylated tau protein and senile plaques containing amyloid β-protein (Aβ; Lloret et al., 2015). The etiology and pathogenesis of the disease is currently not well understood, and management is mostly symptomatic, aimed at ameliorating the cognitive deficits (Selkoe et al., 2012). Furthermore, a recent meta-analysis has shown that relatively few clinical trials are being undertaken for AD therapeutics, and the number of compounds progressing to regulatory review is among the lowest found in any therapeutic area (Cummings et al., 2014), highlighting the importance of developing new therapeutic approaches in this area.

### Fatty Acid Composition

Several studies have investigated the relationship between blood EPA, DPA and DHA levels and risk of cognitive decline. Higher plasma EPA, but not DHA, was found to be associated with lower gray matter atrophy of the hippocampal/parahippocampal area and amygdala of community dwellers aged 65 years and older (Samieri et al., 2012), and higher plasma EPA, but not DPA or DHA, was also associated with slower cognitive decline (Samieri et al., 2011), dementia risk (Samieri et al., 2008) and depressive symptom risk in the elderly (Feart et al., 2008). Furthermore, a recent meta-analysis found that although blood EPA, DHA and total omega-3 PUFA are significantly decreased in patients with dementia, only EPA was significantly lower in patients with pre-dementia syndrome, leading the authors to suggest that EPA may by a biomarker and indeed risk factor for age-related cognitive impairment (Lin et al., 2012a). However, others have shown a reduced risk of developing all-cause dementia associated with higher plasma DHA, not EPA (Schaefer et al., 2006). In this study those in the top quartile of plasma PC DHA level were associated with a significant 47% reduction in risk. Furthermore, a recent cross-sectional analysis of 1575 dementia-free participants also found only lower erythrocyte DHA, but not EPA, associated with significantly smaller brain volumes and lower scores in tests of visual memory, executive function and abstract thinking (Tan et al., 2012). These inconsistencies between observations are likely due to the issues described above limiting the efficacy of using blood fatty acid levels as a surrogate biomarker for CNS levels.

In order to address some of these issues, a recent innovative study investigated the differences in fatty acid content of matched plasma and brain cortex samples from post-mortem samples from subjects with no cognitive impairment, mild cognitive impairment and AD (Cunnane et al., 2012). To date this has been the only study to directly compare plasma and brain levels from the same subjects. In the AD group DHA, but not EPA or DPA, was found to be higher in the plasma cholesteryl esters and lower in plasma phospholipids. In the brain, DHA was lower only in PS of the mid-frontal cortex and superior temporal cortex of AD subjects compared to non-cognitively impaired. Interestingly, there was only one significant correlation between plasma and brain fatty acid samples and this was between DHA in plasma total lipids and DHA in PE of the angular gyrus, but this was only seen in the subjects with mild cognitive impairment. Overall, these results suggest that the fatty acid content of plasma from subjects with mild cognitive impairment or AD may not reflect the content of the brain cortex.

Astatrita and coworkers investigated the role of liver DHA biosynthesis in the altered brain DHA content seen with AD patients (Astarita et al., 2010). Liver DHA content was lower in AD patients than control subjects, whereas EPA, DPA and tetracosahexaenoic acid (24:6n-3), were significantly elevated. Consistent with these changes, expression of peroxisomal _D_-bifunctional protein was reduced; suggesting decreased activity of this enzyme is involved in impaired DHA biosynthesis, thereby lessening the flux to the brain. However, the relative contribution of DHA biosynthsized by the liver compared to that provided by dietary intake remains to be established, as does the overall effect of this on blood DHA levels.

### Inflammation, Learning and Memory

The aging brain is particularly prone to inflammatory and oxidative alterations, which may underlie decreases in learning and memory, as manifested in the age-related deficits in long-term potentiation (LTP; Lynch, 2004). LTP is an electrophysiological property of certain neuronal circuits that is used as a model for investigation of the pathways underlying activity driven neuronal and synaptic plasticity, learning and memory (Bliss and Collingridge, 1993). Dietary enrichment of aged-rats with EPA, DHA and more recently DPA have all been shown to have positive effects on age-related impairments in LTP, and these effects are likely mediated via multiple anti-inflammatory effects acting via alterations in cytokine levels. For example, 8 weeks dietary supplementation with 10 mg/day DHA reverses age-related impairments in LTP and depolarization-induced glutamate release (McGahon et al., 1999). Similarly, feeding rats diets supplemented with EPA for 8 weeks (10 mg/day for 3 weeks and 20 mg/day for 5 weeks) prevented age-related increases in cortical and hippocampal IL-1β and restores LTP (Martin et al., 2002), age-related increases in IL-1β-induced signaling and decreases in IL-4, and extracellular-signal-regulated kinases (ERK) and PI-3 kinase (Maher et al., 2004). 125 mg/day EPA for 4 weeks also attenuated age-related increases in hippocampal IL-1β, interferon-γ, and decreases in IL-4 (Lynch et al., 2007). EPA also protects aged rats from amyloid-β (Aβ) induced increases in hippocampal IL-1β, potentially mediated by positive effects on the PPARγ nuclear transcription factor (Minogue et al., 2007). This group also recently compared the effects of DPA with EPA, where similar neuroprotective effects were reported (Kelly et al., 2011). In this study both DPA and EPA (200 mg/kg/day) were equally potent at reversing age-related impairment in spatial learning and LTP, and showed similar abilities to decreases in age-related microglial activation and associated oxidative stress.

Significant advances have been made in our understanding of the role of DHA and DHA-derived anti-inflammatory mediators such as neuroprotection (NPD-1) in brain cell survival and repair in the aging and AD brain (e.g., Bazan et al., 2011a). However, few studies have made direct comparisons with EPA or DPA or their metabolites. A recent study has however compared the relative anti-inflammatory effects of EPA and DHA *in vitro* in peripheral blood mononuclear cells from AD patients compared with healthy controls (Serini et al., 2012). The addition of either EPA or DHA (10–20 µM) significantly reduced the induced-cytokine release. Although DHA consistently showed a more pronounced effect than EPA, DHA reduced only the high IL-1β/IL-10 ratio, whereas EPA also reduced the IL-6/IL-10 ratio. Overall, DHA had a more powerful inhibitory effect on individual inflammatory cytokines; however, EPA was better at changing the proinflammatory profile of the cells from AD patients to a profile closer to that found in the healthy controls.

### Clinical Trials

To date clinical trials with omega-3 PUFAs in healthy older adults, individuals with mild cognitive impairment or AD have tended to use mixed EPA and DHA preparations at different ratios, and have been reviewed extensively elsewhere (Jiao et al., 2014). There have however been a limited number of trials using either highly enriched EPA or DHA preparations. An early open-label pilot study treated AD patients (MMSE scores 10–24) aged 65 and over with 1 g/day EPA treatment for 12 weeks (Boston et al., 2004). This short-term treatment significantly increased erythrocyte EPA and DPA, but not DHA, and there was no difference between treatment and baseline scores in cognitive measures. In a more recent randomized-blind, placebo-controlled study participants with mild to moderate AD (MMSE scores 14–26) were supplemented with 2 g/day DHA for 18 months (Quinn et al., 2010). The DHA supplementation did not slow the rate of cognitive decline compared to the placebo, although there may have been weak effects with ApoE-ε4 non-carriers.

More positive effects have been seen in cognitively healthy participants and participants with mild cognitive impairment. Yurko-Mauro and colleagues supplemented healthy adults aged 55 years and over with MMSE scores above 26 with 900 mg DHA or placebo for 24 weeks (Yurko-Mauro et al., 2010). The treatment significantly improved Paired Associate Learning (PAL, six pattern errors) and immediate and delayed Verbal Recognition Memory scores, and plasma DHA levels doubled and correlated with improved PAL scores in the DHA group. Similarly, participants with mild cognitive impairment and aged 60 years and over were given either 1.3 g DHA and 0.45 mg EPA or placebo for 12 months and those in the DHA treatment group had significant improvements in short-term and working memory, immediate verbal memory and delayed recall memory compared to the placebo group (Lee et al., 2013). These results are consistent with a study showing positive effects in a sub-group of patients with very mild cognitive impairment (MMSE > 27) who were given a high DHA supplement (1.7 g DHA and 0.6 g EPA) for 6 months (Freund-Levi et al., 2006).

There has only been one study to directly compare the effects of DHA with EPA in participants with mild cognitive impairment (Sinn et al., 2012). In this 6 month double-blind randomized controlled trial participants received a supplement high in EPA (1.67 g EPA and 0.16 g DHA), high in DHA (1.55 g DHA and 0.40 g DHA) or high in the omega-6 PUFA linoleic acid (2.2 g). Compared to the linoleic acid group both the EPA and DHA groups significantly improved their Geriatric Depression Scores, but only with the DHA group were changes memory seen, where there were significant improvements in verbal fluency.

Overall, where individual omega-3 PUFAs have been investigated in trials of healthy aging, mild cognitive impairment and AD the focus has been on DHA, which is consistent with the pre-clinical evidence (Dyall and Michael-Titus, 2008). DHA treatment appears to show the greatest promise compared to EPA and DPA, and the beneficial of DHA treatment currently appear to be to improve memory and learning in participants with MMSE scores above 26–27, particularly non-ApoE-ε4 carriers, although support from clinical trials is currently extremely limited.

## Parkinson’s Disease

Parkinson’s disease (PD) is the second most common neurodegenerative disorder after AD and has a prevalence of about 2% in the older person, and is also associated with aging (Franco-Iborra et al., 2015). PD affects the basal ganglia with patients typically experiencing slowed movements (bradykinesia), muscle stiffness (rigidity), tremor and disturbed balance (Franco-Iborra et al., 2015). The neuropathology of the disease includes dopaminergic neuronal loss from the substantia nigra pars compacta as well as the presence of Lewy bodies, which are intracellular inclusions largely composed of the protein, α-synuclein (Kim et al., 2014b). The pathology and clinical features of PD and AD are markedly different; however, they do share common mechanism, including mitochondrial dysfunction (Camilleri and Vassallo, 2014), neuro-inflammation (Sanchez-Guajardo et al., 2015), and oxidative stress (Xie et al., 2014). Current symptomatic treatment of PD is based on correcting dopaminergic signaling, using medications such as carbidopa/levodopa, dopamine agonists. However, a large number of patients develop mild to moderate, and occasionally serious, treatment-associated side-effects, significantly limiting the efficacy of these treatments (Faulkner, 2014).

### Fatty Acid Composition

Purified lipid rafts from the frontal cortex of postmortem samples from patients with early motor stage PD and incidental PD exhibit significant reductions in DHA (and AA), but not EPA or DPA, compared to controls (Fabelo et al., 2011). Whereas, no differences in the levels of EPA, DPA or DHA were found in post-mortem analysis of temporal cortex tissue from levodopa-treated PD patients, or monkeys treated with the Parkinsonian neurotoxin 1-methyl-4-phenyl-1, 2, 3, 6-tetrahydropyridine (MPTP), compared to their respective matched controls (Julien et al., 2006). However, significant decreases in DHA were seen in the temporal cortical fatty acid profile of primates treated with the MPTP following dyskinesiogenic levodopa treatment, suggesting the decrease in DHA may be related to the appearance of dyskinesia (Julien et al., 2006).

### Pre-Clinical Studies

Although there have to date been no clinical trials reported with supplementation of highly enriched EPA, DPA or DHA in the treatment PD there have been a number of *in vitro* and *in vivo* studies. Mice were fed for 6 weeks a diet providing 0.8% EPA, and their brain slices treated with the Parkinsonian neurotoxin 1-methyl-4-phenylpyridinium (MPP(+)) (Meng et al., 2010). EPA treatment attenuated the MPP(+) induced increase in AA content, partially attenuated the striatal dopaminergic turnover, and prevented the increases of the pro-apoptotic bax and caspase-3 mRNAs. The treatment significantly increased tissue EPA and DPA, but not DHA levels, suggesting the effects may have been mediated directly by EPA and/or DPA. Following the same dietary paradigm the same group investigated the effects of EPA in the (MPTP)-probenecid (MPTP-P) mouse model of PD (Luchtman et al., 2012). EPA treatment attenuated the MPTP-P induced hypokinesia, ameliorated the procedural memory deficit and also suppressed the production of striatal pro-inflammatory cytokines. However, EPA did not prevent nigrostriatal dopamine loss. The mechanisms behind these neuroprotective effects of EPA were explored *in vitro* by the same group (Luchtman et al., 2013). In MPP(+) treated cells, EPA was shown to attenuate the reduction in cell viability and prevent the presence of cytoplasmic inclusions. EPA treatment also had widespread protective effects, such as attenuating the MPP(+)-induced increase in Tyrosine-related kinase B (TrkB) receptors, and down-regulated reactive oxygen species and nitric oxide, an effect potentially mediated by inhibition of neuronal NADPH oxidase and COX-2. EPA also attenuated an increase in the bax:bcl-2 ratio, and cytochrome c release. The effects of DHA have been explored in MPTP monkeys, where DHA (100 mg/kg) reduced dyskinesias induced by levodopa without affecting the anti-Parkinsonian effects, suggesting DHA may delay the development dyskinesias induced by levodopa, or at least reduce their severity (Samadi et al., 2006). Protective effects of DHA have also been shown in mice given a high DHA diet (5.3 g/kg DHA) and then treated with MPTP (Bousquet et al., 2008). Following the DHA treatment the dopamine levels were preserved and the substantia nigral dopaminergic neurons were protected against losses compared to the controls; however, motor behavior was not measured.

It should however be noted that DHA treatment may potentially increase α-synuclein aggregation and the possible formation of cytotoxic oligomers (De Franceschi et al., 2011; Yakunin et al., 2012). α-Synuclein is a neuronal protein that accumulates progressively in PD and related synucleinopathies. In models systems DHA readily promotes α-synuclein aggregation in *Escherichia coli* cells transfected with the human α-synuclein (De Franceschi et al., 2011). The α-synuclein oligomers were more toxic if generated in the presence of DHA in dopaminergic neuronal cell lines. Furthermore, transgenic mice with the PD-causing A53T α-Syn mutation fed a diet enriched with 0.69% DHA showed increased accumulation of soluble and insoluble neuronal α-synuclein, neuritic injury and astrocytosis (Yakunin et al., 2012). These α-synuclein oligomers have also been shown alter membrane permeability in cellular membrane-mimetic and cell model systems (Fecchio et al., 2013). Further *in vitro* analysis suggests that the enhanced α-synuclein oligomerisation in response to DHA is mediated via the retinoic X receptor (RXR). DHA is an endogenous ligand of RXRs (de Urquiza et al., 2000; Egea et al., 2002; Lengqvist et al., 2004) and aged rats treated with DHA show enhanced RXR expression (Dyall et al., 2010). Moreover, Lengqvist and coworkers have shown that other PUFAs such as DPAn-6 and AA are able to bind and activate RXR in the same range as DHA, indicating RXR activation is not restricted to DHA, but may also extend to EPA and DPA.

Overall, there is a lack of clinical trials investigating the role of EPA, DPA or DHA in PD, although pre-clinical evidence suggests that EPA may possess therapeutic potential, and may provide useful adjunctive treatments by alleviating the treatment-associated dyskinesias. However, a 12 week double-blind placebo controlled study providing 2 g/day EPA in schizophrenia patients with established tardive dyskinesia failed to demonstrate any anti-dyskinetic effects, although modest and transient benefits were seen in recent onset patients (Emsley et al., 2006). A clinical trial is currently exploring the role of DHA in reducing dyskinesia in PD (ClinicalTrials.gov identifier: NCT01563913), and should help clarify this issue with DHA at least. It is also important to appreciate that DHA supplementation may have the potential to worsen the condition. The cytotoxic α-synuclein aggregating induced by DHA in a variety of models suggests that caution should be exerted when considering the role of EPA, DPA and DHA in PD and this area warrants much further research.

## Conclusions

This article has reviewed evidence for individual and shared neuroprotective effects of EPA, DPA and DHA in aging and neurodegenerative disorders. EPA, DPA and DHA differ in important aspects of their biochemistry and metabolism; however, few studies have made direct comparisons between their effects. DHA is quantitatively the most important omega-3 PUFA in the brain and has consistently been shown to have unique and indispensable roles in the neuronal membrane. However, independent effects for EPA and DPA are being identified, particularly in regards to their respective anti-inflammatory mediators. A more detailed characterization of the individual properties of these mediators, their cognate receptors and downstream targets may prove essential to increasing understanding of their specific actions.

Deficits in EPA and DHA levels have been found with neurodegenerative disorders; however, these have been identified with varying consistency across studies, and may require a more detailed appreciation of the role of genetic variability before a clear picture arises. Although promising therapeutically, direct evidence from large well-designed intervention studies into the effects of EPA, DPA and DHA in normal aging and neurodegenerative disorders is still lacking; however, preliminary evidence suggests the greatest benefit may been seen with DHA in non-cognitively impaired older people. It is vital to consider omega-3 PUFA specific effects when designing and undertaking systematic reviews and meta-analyses, so treatment effects are not lost in the aggregation of results. Overall, a greater understanding of the individual roles of EPA, DPA and DHA in brain health, protection and repair is needed in order to make appropriate dietary recommendations and targeted therapeutic interventions.

## Conflict of Interest Statement

The author declares that the research was conducted in the absence of any commercial or financial relationships that could be construed as a potential conflict of interest.
